# Neural dynamics of mindfulness training: A longitudinal EEG network analysis of focused attention and open monitoring meditation

**DOI:** 10.1162/NETN.a.555

**Published:** 2026-07-27

**Authors:** Yanli Lin, Marne L. White, Jihong Zhang, Todd S. Braver

**Affiliations:** Department of Psychological Science, University of Arkansas, Fayetteville, AR, USA; Department of Psychology, University of Maryland, College Park, MD, USA; Department of Counseling, Leadership, and Research Methods, University of Arkansas, Fayetteville, AR, USA; Department of Psychological and Brain Sciences, Washington University in St. Louis, St. Louis, MO, USA

**Keywords:** Mindfulness, EEG, Network analysis, Alpha, Theta

## Abstract

Neural oscillatory activity within the alpha and theta bands have long been considered putative markers of state mindfulness, yet understanding of their functional role has been limited by the challenges of linking objective brain indices with subjective experience. To address this gap, the current study applied longitudinal network analysis to a unique dataset from 16 novices who completed up to 24 laboratory training sessions of both focused attention (FA) and open monitoring (OM) meditation, during which both EEG and self-report measures of state mindfulness quality were collected. This approach enabled the parsimonious characterization of both cross-lagged temporal (across-session) and contemporaneous (within-session) influences of regional spectral power on state mindfulness. The analysis revealed distinguishable neurophenomenological network structures for each practice, providing data-driven support for their theoretical differentiation. These distinctions emerged alongside shared commonalities to both practices, including strong autoregressive effects for state mindfulness, consistent with training-related skill acquisition, and opposing regional influences of frontal versus posterior alpha power. Taken together, these findings challenge monolithic interpretations of meditation-related EEG activity, advancing a more nuanced neurophenomenological approach wherein the functional significance of neural activity is dynamically situated within the specific type and time course of training.

## Author Summary

Mindfulness training is increasingly incorporated into science, medicine, and education, yet the neural mechanisms linking subjective mindfulness experience with brain activity remain poorly understood. Using an intensive longitudinal EEG design and network modeling, this study demonstrates the neural dynamics that differentiate focused attention and open monitoring, two foundational mindfulness meditation practices. The findings highlight how dense-sampling and network analytic approaches can capture evolving brain–mind relationships over time, advancing understanding of neurocognitive plasticity within the context of contemplative mental training.

## INTRODUCTION

For over 6 decades, electroencephalography (EEG) spectral analysis has served as a primary tool for investigating the neural underpinnings of contemplative meditation practices (see Cahn & Polich, 2006, for a historical overview). Within this expansive body of literature, neural oscillatory activity occurring within the alpha (8–13 Hz) and theta (4–8 Hz) frequency ranges (i.e., alpha and theta power) has emerged as two particularly prominent “neural signatures” of the mindfulness state: the quality of nonjudgmental awareness and acceptance of present moment experience (Bishop et al., 2004;Kabat-Zinn, 1990), which has become a defining feature of the namesake family of mindfulness meditation and mindfulness-based interventions (Baer, 2003;Hölzel et al., 2011). From a basic cognitive neuroscience perspective, alpha power has been functionally linked to cortical inhibition mechanisms and subjective states of vigilance and internally directed attention (Başar, 2012;Bazanova & Vernon, 2014;Klimesch, 2012), while theta power has been associated with higher order cognitive functions, including memory, motivation, and executive control processes (Cavanagh & Frank, 2014;Herweg et al., 2020;McFerren et al., 2021).

In the context of mindfulness, increased modulation of alpha and theta band power observed across numerous studies have led to the pervasive interpretation that elevated alpha and theta activity may serve as both a trait-level marker of mindfulness among advanced practitioners, as well as indexing a state of “relaxed alertness” arising from meditation (Britton et al., 2014;Cahn & Polich, 2006;Fell et al., 2010;Lee et al., 2018;Lieberman et al., 2025;Lomas et al., 2015). Collectively, this convergence of research has fostered the compelling suggestion that alpha and theta activity patterns may constitute reliable neurophysiological markers of state mindfulness, potentially representing a neurophenomenological bridge between objective brain activity and subjective first-person experience (Berkovich-Ohana et al., 2020;Lutz & Thompson, 2003;Varela, 1996).

Despite the historical prevalence and intuitive appeal of this view, a closer examination of the literature reveals inconsistencies, limitations, and surprisingly tenuous evidentiary support, suggesting that the role of alpha and theta activity as neural markers of state mindfulness requires further empirical substantiation. Importantly, much of the challenge stems from the considerable methodological heterogeneity across studies. Notably, research has varied widely in the specific type of mindfulness practice investigated (e.g., focused attention [FA] vs. open monitoring [OM]; Lutz et al., 2008), study design (e.g., cross-sectional vs. longitudinal; between-subject vs. within-subject), the nature of experimental comparison or control conditions (e.g., active task vs. passive rest), and participant sample characteristics (e.g., novices vs. experts).

To briefly illustrate, across the 28 studies reporting alpha-band metrics included inLomas et al.’s (2015) systematic review, investigation spanned at least 10 different meditation techniques, eight different practice durations, and five different baseline conditions, with 10 studies featuring novice participants, whereas the remaining 18 studies involved advanced practitioners. Moreover, these studies differed extensively with respect to task and analytic procedures, ranging from single session comparisons of alpha power between meditation and rest (Lagopoulos et al., 2009), oddball paradigms contrasting stimulus-locked alpha activity between meditation and mind wandering conditions (Cahn et al., 2013), to longitudinal intensive retreat designs evaluating pre–post changes in individual alpha frequency (Saggar et al., 2012). Critically, this methodological variability likely contributes to the mixed findings being reported, particularly with regard to the directionality, magnitude, spatial topography, and underlying boundary conditions of alpha and theta effects, thereby impeding the development of a coherent theoretical framework with clear generalizable conclusions.

A second major limitation of the prior literature pertains to the frequent disconnect between neural measurement and subjective experience. Most studies to date have assumed the presence or quality of a mindful state based solely on observed changes in alpha and theta activity (e.g., comparing modulation during meditation periods relative to rest). As many others have convincingly outlined, this practice is quite susceptible to reverse inference (Poldrack, 2006;Van Horn & Poldrack, 2009) and related logical fallacies (Bennett & Hacker, 2022), when the engagement of a subjective mental state or execution of a psychological function is inferred solely from brain activity. Without concurrent subjective state measures, attributing changes in EEG activity to the cultivation of mindfulness, even during highly experimentally controlled conditions, remains fundamentally speculative. Consequently, to rigorously establish alpha and theta power as neural*correlates* of state mindfulness, it seems imperative to demonstrate a systematic relationship between variability in these neural indices with corresponding changes in self-reported state mindfulness quality.

The main aim of the current study is to take this important step to clarify the roles of alpha and theta power as putative neural indices of state mindfulness within the specific context of differentiating FA and OM meditation training (Lutz et al., 2008). Notably, FA meditation, which involves sustaining attention on a target object (e.g., the breath), and OM meditation, characterized by a more diffuse nonreactive awareness of momentary experience (e.g., thoughts, feelings, bodily sensations), are two core practices taught within popular mindfulness-based interventions and digital training programs (Britton et al., 2018;Wolff & Beste, 2020). Although emerging evidence indicates that FA and OM may indeed be neurally and functionally distinguishable (Brown et al., 2022;Fox et al., 2016;Lin, White, Viravan, et al., 2024;Lin, White, Wu, et al., 2024), few studies have explicitly investigated the link between alpha and theta dynamics in relation to subjective measures of state mindfulness during and across FA and OM practice. Moreover, because the specific cognitive processes engaged during FA versus OM are likely supported by distinct neural circuits, the topographical distribution of alpha and theta activity across different scalp regions (e.g., frontal, temporal-central, posterior) may also carry important information for distinguishing the nature of these meditative states, yet have been infrequently measured and remain in need of systematic analysis. To begin filling these knowledge gaps, our group recently leveraged a cross-over state induction design to demonstrate that the relationship between spectral power and subjective experience is indeed dissociable across FA and OM states among novices (White et al., 2025). However, the static design cannot determine whether these brain–mind relationships are relatively stable or if they dynamically evolve with practice and experience.

To address this fundamental question, we leverage data from the recently completed Intensive Longitudinal Mindfulness (ILM) project (White et al., 2024), which, to the best of our knowledge, represents the very first study to implement a fully within-subject longitudinal design, wherein both EEG (including regionally differentiated alpha and theta power) and self-report measures of FA and OM training are sampled repeatedly within the same participants as they completed*both* practices across many meditation sessions. Briefly, participants completed 8 weeks of FA and OM training involving up to 24 EEG-recorded laboratory practice sessions (three visits per week), during which they completed 20 min of standardized audio-guided FA and OM meditation practice (totaling 40 min of practice per session). Following each meditation, participants provided self-report ratings on the quality of their respective practice using the validated State Mindfulness Scale (SMS;Tanay & Bernstein, 2013) and other targeted single-item measures (seeWhite et al., 2024, for the full study protocol).

Crucially, this intensive repeated sampling protocol, pairing EEG recordings of FA and OM meditation with temporally proximal self-reports of subjective mindfulness quality across recurring training sessions, enables analysis to move beyond the limitations of static cross-sectional or traditional pre–post comparison designs. By directly modeling both alpha and theta power across frontal, temporal-central, and posterior scalp regions as predictors of corresponding state mindfulness ratings across sessions, this within-subject approach elucidates whether intra-individual dynamic variations in these neural signals are meaningfully associated with variability in the subjective quality of FA and OM practice over the course of training. Here, we conducted a rigorous test for the presence of such within-subject relationships, with the goal of obtaining evidence to support the long-held proposition that alpha and theta activity index neural correlates of state mindfulness. Although other frequency bands have also been implicated in contemplative neuroscience (e.g., gamma, beta), we intentionally focused the scope of this initial investigation on alpha and theta. This decision was guided by their historical prominence and established links to core cognitive and attentional processes central to both FA and OM (Cahn & Polich, 2006;Lee et al., 2018;Lomas et al., 2015), which also served to maintain analytic tractability for this proof-of-concept study.

Toward these ends, we sought to capitalize on the time-intensive design of the ILM study by utilizing advanced longitudinal network analysis (Epskamp, 2020;Epskamp, Borsboom, et al., 2018;Epskamp, Waldorp, et al., 2018). Although viable, standard approaches such as multilevel regression are not well optimized to fully capture both within- and cross-session patterns of brain state relationships as they unfold across time. By leveraging techniques like vector autoregression (VAR) and its multilevel applications (i.e., mlVAR), longitudinal network analysis provides an incredibly powerful analytic framework to move beyond estimating “static” within-session associations among a constrained number of predictors (e.g., strength of partial correlation between mindfulness and alpha power across sessions). Instead, the approach models region-specific alpha and theta power and state mindfulness ratings as separate nodes within a unified dynamical network, wherein the edges between nodes represent directional statistical relationships over time.

Consequently, longitudinal network analysis offers several key unique advantages that maximize inferential ability to remediate the aforementioned gaps and limitations of prior research. First, the approach can handle many theoretically relevant variables simultaneously, such as alpha and theta power, separated over distinct frontal, temporal-central, and posterior topographic scalp regions, and state mindfulness scores differentiated across mind and body subscales. Second, the models can estimate not only contemporaneous relationships (i.e., how variables relate to each other within a session) but also autoregressive and cross-lagged effects (i.e., how one variable influences itself and other variables*across* sessions). Third, network analysis allows for the estimation of node centrality metrics, which quantify the relative importance or influence of each node within the overall network structure. Identifying influential nodes (e.g., region specific band power, or particular aspects of subjective mindfulness) can illuminate key drivers of the system’s dynamics and pinpoint potent targets that modulate theneurophenomenology of meditation training. Together, these analytic features enable unprecedented characterization of how region-specific alpha and theta neural oscillatory activity interact dynamically to modulate subjective state mindfulness over the course of meditation training.

Accordingly, the primary aim of this initial study was to fully leverage the unique data structure of the ILM project by applying longitudinal network analysis to test the intuitive yet undertested theoretical hypothesis that training-induced changes in neural oscillatory activity during meditation would prospectively enhance subjective state mindfulness quality. In light of growing evidence supporting the distinct nature of FA and OM meditation practice, a second aim of the study was to test whether the dynamic relationship between neural oscillatory activity and state mindfulness was distinguishable across the two practices. To investigate this question, separate longitudinal networks were conducted for FA and OM training data to directly compare and differentiate the neurophenomenological network structure of FA and OM.

## METHODS

### Participants

Participants were recruited from the Washington University in St. Louis community and surrounding areas. After screening and consent procedures, 19 individuals enrolled in the study and initiated the training protocol. Among this group, 16 participants finished the full study protocol, completing an average of 22.81 training sessions out of the 24 total sessions (95.04%). The remaining 3 participants voluntarily discontinued during or prior to the midpoint of training (after 12 sessions, 6 sessions, and 4 sessions, respectively) and were thus excluded from the analyses due to data sparsity (see theStatistical Analyses section for further details). Consequently, the final sample consisted of 16 participants with the following demographics: 11 White; 3 Asian; 1 Black/African American; 1 more than one race; 2 Hispanic/Latino (mean age = 41.06 years,*SD* = 23.62 years; 8 female, 7 male, 1 nonbinary). Nine participants completed all 24 laboratory training sessions, two participants completed 23 sessions, one participant completed 22 sessions, three participants completed 21 sessions, and one participant completed 18 sessions. Participants were fluent English speakers, aged between 18 and 85 years, and reported no history of severe neurological illness or injury. All participants provided informed consent prior to enrollment and received monetary compensation for their participation. The study protocol was approved by Washington University in St. Louis’ Institutional Review Board (202206156).

### Procedures

The ILM study consisted of an 8-week training period, combined with pre- and posttraining assessments. For a comprehensive description of the full ILM study, including additional recruitment details and all assessment protocols and measures, seeWhite et al. (2024). To circumscribe the scope of the current report, we focus on only the relevant procedures and materials from the training phase of the study; forthcoming papers will feature the pre- and postassessment data. During the training period, participants attended up to 3 EEG-recorded laboratory practice sessions per week, totaling a maximum of 24 sessions. Each laboratory session involved participants completing two alternating 10-min blocks of standardized audio-guided FA and OM meditation practices (i.e., FA-OM-FA-OM or OM-FA-OM-FA), resulting in 20 min of FA and OM practice each (40 min of meditation total). Continuous EEG activity was recorded throughout each meditation block. Immediately following the second iteration of each practice (i.e., after the second and fourth rounds of meditation), participants completed a self-report questionnaire assessing the subjective quality of their just-completed practice.

### Tasks and Materials

#### Audio-guided meditations.

The FA and OM guided meditations were both recorded by a certified Mindfulness-Based Stress Reduction teacher and have been successfully implemented in prior work, including empirical differentiation of their effects on cognition and emotion (Lin, White, Viravan, et al., 2024;Lin, White, Wu, et al., 2024;Tang & Braver, 2020). The FA meditation recording instructed participants to sustain attention on the breath, while the OM recording guided them to direct awareness to arising momentary experience (e.g., thoughts, feelings, sensations) with acceptance and nonjudgment. Both meditations were duration matched at 10 min in length and presented using E-Prime software (Psychology Software Tools Inc., Sharpsburg, PA, USA), programmed with EEG trigger codes sent every minute to ensure timing synchronization of the EEG recording. For standardization and to minimize the known influence of fatigue and sleepiness on EEG activity, all participants were seated upright in a chair and instructed to keep their eyes open during the practice.

#### SMS.

As noted above, participants completed the 21-item State Mindfulness Scale (SMS;Tanay & Bernstein, 2013) immediately after the second round of FA and OM practice during each session (i.e., completing the SMS twice, once following FA and once after OM). Participants were instructed to rate the subjective quality of each practice by rating their level of awareness across various experiential dimensions on a 5-point Likert scale ranging from 1 (*not at all*) to 5 (*very well*), differentiated by mindfulness of mind (e.g., thoughts, emotions, mental acuity; SMS-Mind) and mindfulness of body (e.g., physical and bodily sensations; SMS-Body) subscales.

### EEG Recording and Preprocessing

Participants were fit with a 64-channel Lycra cap, and a Brain Vision actiCHamp Plus system was used to record EEG activity (Brain Vision LLC, Morrisville, NC). Recordings were obtained from 32 Ag-AgCl electrodes arranged in the 10/20 system with a sampling rate of 500 Hz. Three electrodes were placed around the eyes to capture horizontal and vertical electrooculogram activity, two lateral to the outer canthi of each eye and one under the right pupil below electrode site Fp2.

Offline preprocessing was completed using BrainVision Analyzer 2 (BrainProducts, Gilching, Germany). All data were first rereferenced to the average of all scalp electrodes (i.e., common average reference), band-pass filtered between 0.3 and 50 Hz, and subjected to regression-based ocular artifact correction (Gratton et al., 1983). The data were then segmented into 2-sec epochs and subsequently subjected to automatic artifact rejection based on the following criteria: a maximum voltage step of more than 50*μ*V between sample points, a voltage difference of more than 300*μ*V within 200-msec intervals, voltage exceeding ±200*μ*V, or a maximum voltage difference less than 0.5*μ*V within 1,000-msec intervals. A fast Fourier transform was applied to all retained artifact-free epochs, weighted with a hamming window tapering the distal 10% of each epoch, and integrated spectral power was computed for the alpha (8–13 Hz) and theta (4–8 Hz) frequency bands.

Drawing from previous work (e.g.,Lagopoulos et al., 2009;Lin et al., 2020), power values at each electrode site were averaged across three scalp regions: frontal (Fp1, Fpz, Fz, F3, F7, FC1, FC5, FC2, FC6, F4, F8, Fp2), temporal-central (C3, CP1, CP5, CP2, CP6, C4), and posterior (Oz, P10, P4, P3, P9, PO3, PO4, PO7, PO8, Pz). As standard, all values were log transformed to normalize their distribution prior to analyses.

### Statistical Analysis

Longitudinal network analysis was employed to investigate the dynamic interrelations between neural (alpha and theta power) and subjective (SMS-Mind and SMS-Body scores) measures of meditation quality over the course of FA and OM training. For each of the FA and OM networks, a total of eight nodes comprising alpha and theta power separated across respective frontal, temporal-central, and posterior regions (frontal alpha, frontal theta, temporal-central alpha, temporal-central theta, posterior alpha, posterior theta), and SMS-Mind and SMS-Body scores were included. To handle session-level missingness arising from participants not completing all 24 sessions, the full information maximum likelihood (FIML;Cham et al., 2017) method was utilized to make use of all available data points from each participant without needing to truncate the time series or impute data.

Graphical vector autoregressive (GVAR) models (Wild et al., 2010) were implemented to estimate the network structures using the*psychonetrics* package (Epskamp, 2025) in the R software environment (R Core Team, 2025). Critically, two orthogonal network types were derived to investigate the cross-lagged temporal and contemporaneous interconnections among node variables. Briefly, the cross-lagged network estimated the extent to which a source node at a previous session (*t*–1) predicts a target node at the current session (*t*), while controlling for the effects of all other nodes and the target node’s previous state (i.e., itsautoregressive effect). Consonant with the primary aim of the investigation, we allowed cross-lagged pathways from the EEG nodes at*t*–1 to SMS nodes at*t* to be freely estimated while constraining the cross-lagged pathways from SMS nodes at*t*–1 to EEG nodes at*t* to zero, thereby isolating analysis to the unidirectional predictive influence of brain activity on self-reported mindfulness. Importantly, this constraint was proactively implemented to reduce model complexity and enhance interpretability for this initial proof-of-concept investigation, while maintaining theoretical consistency with our aim of grounding neural dynamics in their subjective context. Thecontemporaneous network estimated the partial correlations (i.e., undirected effects) between nodes within the*same concurrent* session, after controlling for temporal influences. It is important to note that although this network models relationships as contemporaneous at the session level, our study procedures had a finer temporal structure where EEG was recorded prior to administration of the SMS. The GVAR model treats all variables within a discrete time point (i.e., session) as concurrent; thus, our interpretation of the contemporaneous network reflects this modeling assumption.

To maintain network interpretability, nonsignificant edges (adjusted*p* > .05) in all network types were pruned after applying a false discovery rate (FDR) threshold of 0.05 to correct for multiple comparisons. Furthermore, to assess the accuracy and stability of the estimated networks, a nonparametric resampling bootstrapping procedure (1,000 samples) was applied to compute the proportion of bootstrapped networks in which edges retained the same signs as in the original estimated network (i.e., an edge exhibiting high probability of retaining the same directionality should possess high stability;Epskamp, Borsboom, et al., 2018).

Finally, to evaluate node influence within the cross-lagged networks, we computed the following centrality metrics based on the standardized partial directed correlation (PDC) matrix derived from the temporal GVAR model (rather than unstandardized regression coefficients), consistent with prior GVAR applications: (a)*InStrength*, quantified as the sum of all*incoming* edge weights to a node, reflective of how much a node is influenced by other nodes at previous time points; (b)*OutStrength*, quantified as the sum of all*outgoing* edge weights from a node, reflective of how much a node influences other nodes in future time points. For the contemporaneous network, we computed: (a)*strength*, quantified as the sum of all undirected edge weights to a node; (b)*closeness*, quantified as the inverse of the sum of the shortest distances from a node to all other nodes in the network, indicating how quickly a node can reach other nodes; (c)*betweenness*, quantified as the sum of times a node lies on the shortest path between other node pairs, indicating the extent to which it connects disparate parts of the network. Note that closeness and betweenness were not presented for thecross-lagged temporal network due to their interpretative ambiguity within directed networks (Hallquist et al., 2021). All data and analytic code, including full model specifications and additional visualizations, are publicly available (https://osf.io/buxah/). For full transparency, we note that the longitudinal network approach represents a methodological deviation from our preregistered aims (https://osf.io/gws3q), which specified the use of linear mixed-effects models. We determined based on the reasons and advantages outlined above that GVAR provided a more powerful and theoretically appropriate analytic framework for modeling the dynamic, time-lagged relationships among the topographically distributed neural indices inherent in our intensive longitudinal data.

## RESULTS

**Figure 1.**
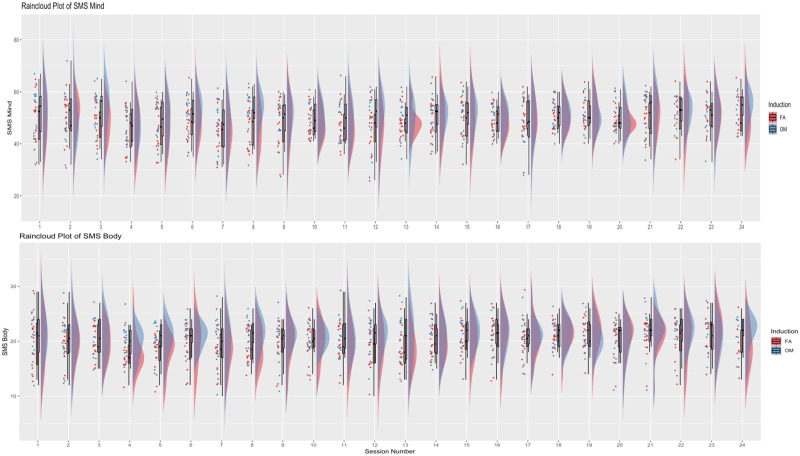
Raincloud plots depicting the distribution of SMS-Mind and SMS-Body scores across sessions. Visual summary of the distribution and session-to-session variability of subjective state mindfulness ratings (SMS-Mind, SMS-Body) across the 24 training sessions. Dots represent individual data points; box plots show the median and interquartile range. Red represents FA; blue represents OM.

Descriptive statistics of SMS scores separated across FA and OM, including distributional changes across training sessions, can be found in Table 1 and Figure 1. Although the primary focus of the present study was to apply longitudinal network modeling to assess dynamic patterns of brain–mind coupling across training, we conducted complementary linear mixed-effects analyses to assess whether subjective mindfulness ratings increased across sessions. Results indicated that both SMS-Mind and SMS-Body scores increased modestly but reliably over the course of training, with higher scores overall during OM relative to FA, but no evidence for differential rates of change across practice. These findings suggest that mindfulness quality improved across sessions, providing contextual support for the longitudinal network analyses conducted here. Full model specifications and output of these supplementary analyses are available via the Open Science Framework (OSF) repository (https://osf.io/buxah/).

**Table 1.** Descriptive statistics of state mindfulness scores

Variable	FA			OM		
	Range	*M*	*SD*	Range	*M*	*SD*
SMS-Total	40–93	68.67	10.24	38–95	71.00	9.88
SMS-Mind	26–66	48.75	7.58	27–72	50.26	7.61
SMS-Body	10–29	19.92	3.68	10–29	20.74	3.34

Below, we report the results from the longitudinal network analyses separately for FA and OM. For each meditation type, we prioritize the description of the key findings from the cross-lagged and contemporaneous networks, specifically focusing on significant brain–mind pathways (i.e., EEG power to SMS scores). To maintain brevity and alignment with the core research question, we refrain from narratively describing each interrelational permutation among the six EEG nodes. Nevertheless, these interbrain dynamics are fully represented visually and numerically across all network models.

### FA Meditation

#### Cross-lagged temporal network.

**Figure 2.**
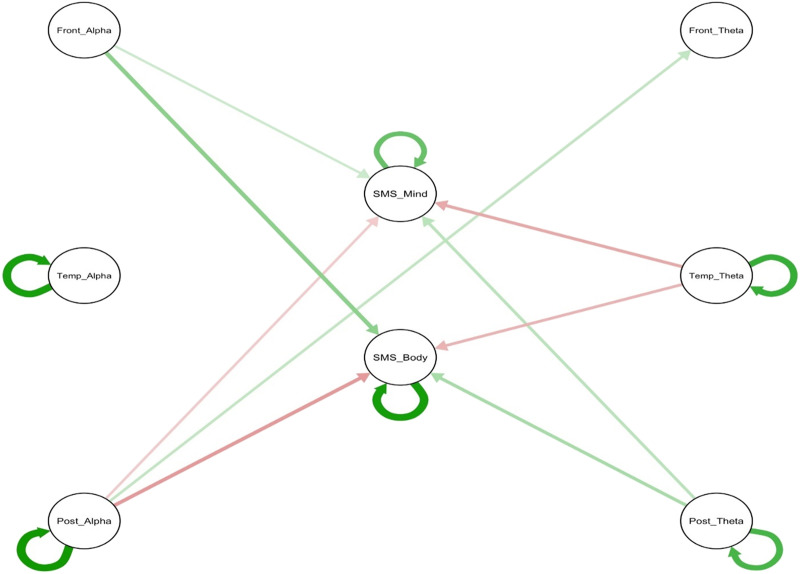
FA cross-lagged temporal network. Graphical depiction of significant cross-lagged associations linking region-specific EEG spectral power (alpha, theta) and subjective state mindfulness ratings (SMS-Mind, SMS-Body) across training sessions of FA practice. Directed edges (arrows) indicate a unique temporal effect from session *t* − 1 to session *t*. Green arrows denote positive associations; red arrows denote negative associations. Self-loops represent autoregressive effects. Line thickness and color intensity reflect the magnitude of the regression coefficient; nonsignificant edges (FDR-adjusted *p* > .05) are pruned.

Supporting the foundational premise that meditation-related brain activity prospectively influences subjective mindfulness experience, the cross-lagged FA network, which demonstrated excellent stability (mean proportion of replicated bootstrapped samples = 0.99), revealed a dynamic pattern of region-specific facilitative and inhibitory effects (see Figure 2 and Table 2). In terms of neural predictors, a bidirectional pattern was observed for alpha power, such that increased frontal alpha power during a given session enhanced awareness of mind (*β* = 0.60, *SE* = 0.30, *p* = .04) and, even more strongly, awareness of body (*β* = 1.30, *SE* = 0.30, *p* < .001) in the next. Conversely, decreased posterior alpha power was subsequently linked to greater mindfulness of both mind (*β* = −0.54, *SE* = 0.22, *p* = .01) and body (*β* = −1.02, *SE* = 0.22, *p* < .001). A similar dynamic was observed for theta power, but with the opposite topography, such that greater posterior theta power during a given practice session enhanced mindful awareness of both mind (*β* = 0.59, *SE* = 0.23, *p* = .01) and body (*β* = 0.73, *SE* = 0.23, *p* = .001) during subsequent sessions, while, in contrast, decreased temporal-central theta power was associated with a next-session increase in state mindfulness of both mind (*β* = −0.80, *SE* = 0.26, *p* = .002) and body (*β* = −0.67, *SE* = 0.26, *p* = .01).

**Table 2.** Cross-lagged temporal network for FA meditation

		Source							
		Temporal theta	Frontal theta	Posterior theta	Frontal alpha	Temporal alpha	Posterior alpha	SMS-Mind	SMS-Body
		*β* (*SE*)	*β* (*SE*)	*β* (*SE*)	*β* (*SE*)	*β* (*SE*)	*β* (*SE*)	*β* (*SE*)	*β* (*SE*)
Targets	Temporal theta	**0.91 (0.24)** **	−0.08 (0.22)	0.04 (0.19)	−0.41 (0.26)	0.16 (0.19)	0.31 (0.17)		
	Frontal theta	0.37 (0.24)	0.33 (0.21)	0.13 (0.19)	−0.33 (0.26)	<0.01	**0.38 (0.18)** *		
	Posterior theta	0.20 (0.24)	−0.10 (0.22)	**0.79 (0.19)** **	−0.37 (0.26)	0.10 (0.19)	0.30 (0.17)		
	Front alpha	0.13 (0.24)	−0.05 (0.22)	−0.06 (0.19)	0.40 (0.26)	0.18 (0.19)	0.33 (0.17)		
	Temporal alpha	0.10 (0.24)	0.05 (0.22)	−0.12 (0.19)	−0.34 (0.26)	**0.99 (0.19)** **	0.26 (0.17)		
	Posterior alpha	0.11 (0.24)	0.04 (0.22)	−0.12 (0.19)	−0.28 (0.26)	0.14 (0.19)	**1.06 (0.17)** **		
	SMS-Mind	**−0.80 (0.26)** **	−0.12 (0.21)	**0.59 (0.23)** *	**0.60 (0.30)** *	0.36 (0.22)	**−0.54 (0.22)** *	**0.25 (0.06)** **	0.09 (0.06)
	SMS-Body	**−0.67 (0.26)** *	−0.24 (0.21)	**0.73 (0.23)** **	**1.30 (0.30)** **	−0.09 (0.21)	**−1.02 (0.22)** **	−0.03 (0.07)	**0.40 (0.06)** **

*Note*. Significant paths are **bolded** for ease of reference. Significance testing is not conducted for *β* values < 0.01.

* *p* < 0.05.

** *p* < 0.01.

Beyond these cross-lagged neural effects, both mindfulness subscales exhibited strong positive autoregressive effects. Higher SMS-Mind scores in one session significantly predicted higher SMS-Mind during the next (*β* = 0.25, *SE* = 0.06, *p* < .001) and likewise higher SMS-Body scores predicted subsequently higher SMS-Body scores (*β* = 0.40, *SE* = 0.06, *p* < .001), providing evidence of a facilitatory feedback loop often assumed to occur during training, wherein cultivating mindfulness strengthens more mindfulness, a hallmark of skill acquisition where prior success facilitates future performance.

The cross-lagged network’s centrality metrics shed further light on these dynamical relationships (seeFigure 3). In particular, SMS-Body (InStrength = 1.97) and SMS-Mind (InStrength = 1.16) were the primary downstream targets, reinforcing the primacy of subjective experience, as these measures were heavily influenced by the combination of prior neural and subjective states. Conversely, posterior alpha exhibited the highest OutStrength (1.34), identifying it as a key network hub that exerts the most influence on the overall state of the network over the course of training. This pattern suggests that although subjective mindfulness quality is the primary outcome of training dynamics, fluctuations in posterior alpha power are a key driver of those session-to-session changes.

**Figure 3.**
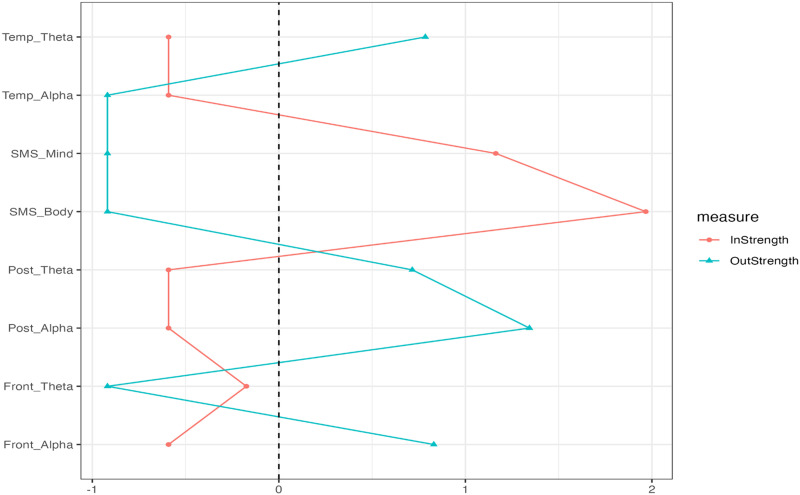
FA cross-lagged temporal network centrality metrics. Plot displaying centrality indices for the FA cross-lagged network. InStrength (red circle) quantifies the sum of incoming edge weights, reflecting the extent to which a node is influenced by others over time. OutStrength (blue triangle) quantifies the sum of outgoing edge weights, reflecting a node’s influence on the future state of the network. Centrality indices are calculated based on standardized partial directed correlations, which differ from the regression coefficients reported in Table 2.

#### Contemporaneous network.

The contemporaneous FA network, which showed strong stability (mean proportion of replicated bootstrapped samples = 0.97), revealed two significant associations involving state mindfulness (seeFigure 4 andTable 3). As expected, SMS-Mind was strongly and positively associated with SMS-Body (*β* = 0.55, *SE* = 0.04, *p* < .001). Interestingly, frontal theta was negatively associated with SMS-Mind (*β* = −0.12, *SE* = 0.06, *p* = .04) but not SMS-Body (*β* = 0.04, *SE* = 0.06, *p* = .50), suggesting that reduced frontal theta power may be a specific neural correlate of mindful awareness of mental activity during FA. Consistent with this notion, centrality analysis of this network (seeFigure 5) revealed that frontal theta was the most influential node, where its high values across multiple indices (betweenness = 2.09, closeness = 1.14, strength = 1.18) position it as a critical hub.

**Figure 4.**
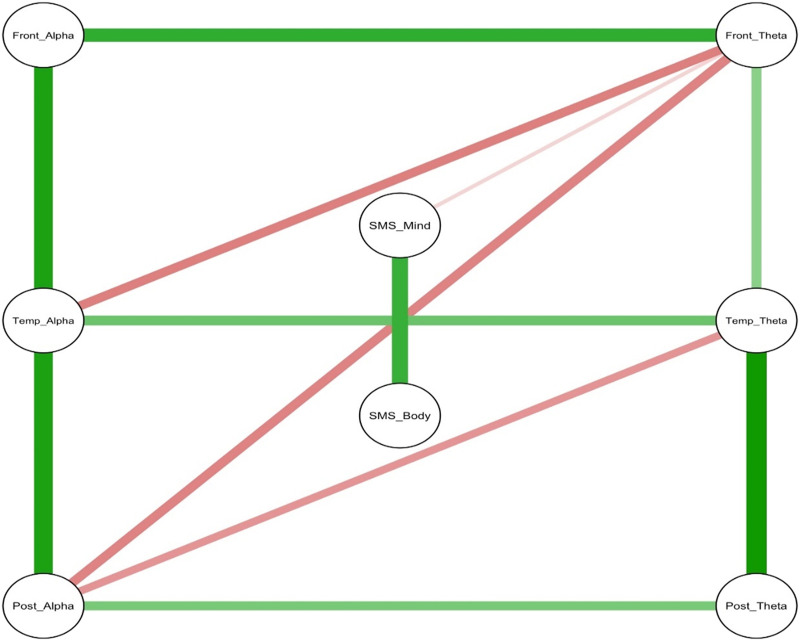
FA contemporaneous network. Graphical depiction of significant within-session partial correlations linking region-specific EEG spectral power (alpha, theta) and subjective state mindfulness ratings (SMS-Mind, SMS-Body) during FA practice. Edges represent undirected associations between variables during the concurrent session *t*. Green edges denote positive partial correlations; red edges denote negative partial correlations. Line thickness and color intensity reflect the magnitude of the association; nonsignificant edges (FDR adjusted *p* > .05) are pruned.

**Table 3.** Contemporaneous network for FA meditation

		Source						
		Temporal theta	Frontal theta	Posterior theta	Frontal alpha	Temporal alpha	Posterior alpha	SMS-Mind
		*β* (*SE*)	*β* (*SE*)	*β* (*SE*)	*β* (*SE*)	*β* (*SE*)	*β* (*SE*)	*β* (*SE*)
Targets	Frontal theta	**0.29 (0.08)** **						
	Posterior theta	**0.71 (0.05)** **	**0.32 (0.08)** **					
	Frontal alpha	−0.04 (0.12)	**0.57 (0.06)** **	−0.18 (0.12)				
	Temporal alpha	**0.41 (0.10)** **	**−0.35 (0.08)** **	−0.10 (0.12)	**0.54 (0.09)** **			
	Posterior alpha	**−0.30 (0.11)** **	**−0.34 (0.09)** **	**0.37 (0.11)** **	**0.65 (0.08)** **	0.20 (0.14)		
	SMS-Mind	−0.03 (0.06)	**−0.12 (0.06)** *	<0.01	0.08 (0.07)	−0.04 (0.07)	−0.02 (0.07)	
	SMS-Body	−0.08 (0.06)	0.04 (0.06)	0.06 (0.06)	<0.01	0.07 (0.06)	−0.06 (0.07)	**0.55 (0.04)** **

*Note*. Significant paths are **bolded** for ease of reference. Significance testing is not conducted for *β* values < 0.01.

* *p* < 0.05.

** *p* < 0.01.

**Figure 5.**
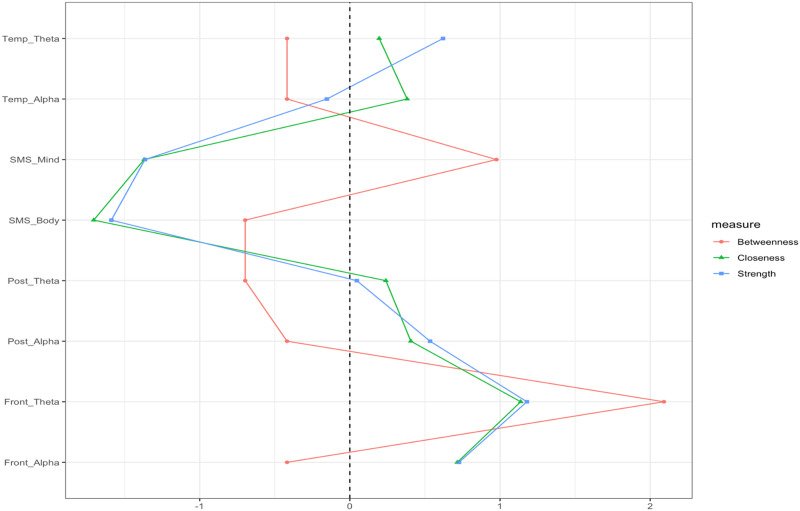
FA contemporaneous network centrality metrics. Plot displaying centrality indices for the FA contemporaneous network. Strength (blue square) represents the sum of absolute edge weights connected to a node, indicating its overall level of direct connectivity. Closeness (green triangle) reflects the inverse of the sum of shortest distances to all other nodes. Betweenness (red circle) indicates how often a node lies on the shortest path between two other nodes. Higher values indicate greater influence within the network.

### OM Meditation

#### Cross-lagged temporal network.

Exemplifying its distinctiveness, the cross-lagged OM network presented a strikingly different and markedly sparser pattern of influences relative to FA (seeFigure 6 and Table 4). The network was also highly stable (mean proportion of replicated bootstrapped samples = 0.97) but revealed fewer predictive neural pathways. Intriguingly, the influence of alpha power was specific to bodily awareness. Higher frontal alpha power during a given session was associated with an increase in SMS-Body (*β* = 0.59, *SE* = 0.28, *p* = .04) during the next, whereas decreased posterior alpha was associated with an increase in SMS-Body (*β* = −0.42, *SE* = 0.19, *p* = .03). On the other hand, the effect of theta power was selective to mindfulness of mind, such that decreased temporal-central theta power predicted a subsequent increase in mindfulness of mind (*β* = −0.64, *SE* = 0.25, *p* = .01).

**Figure 6.**
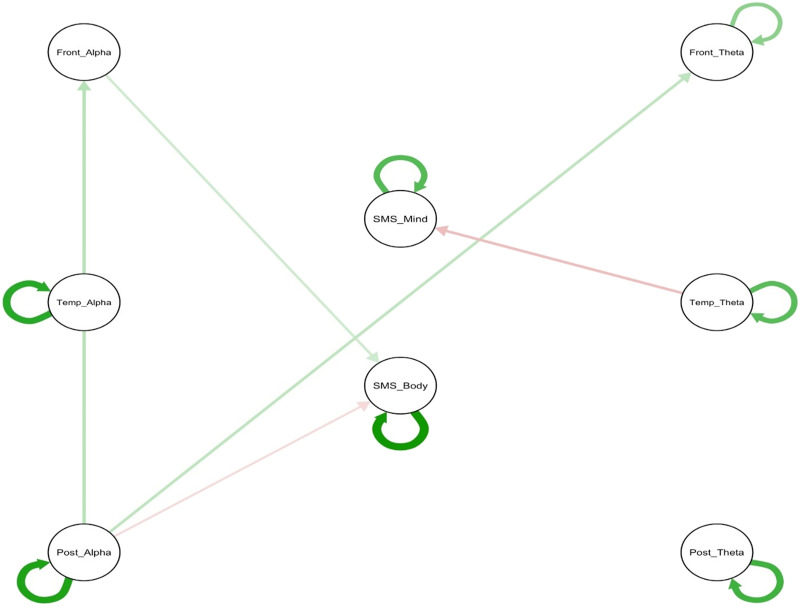
OM cross-lagged temporal network. Graphical depiction of significant cross-lagged associations linking region-specific EEG spectral power (alpha, theta) and subjective state mindfulness ratings (SMS-Mind, SMS-Body) across training sessions of OM practice. Directed edges (arrows) indicate a unique temporal effect from session *t* − 1 to session *t*. Green arrows denote positive associations; red arrows denote negative associations. Self-loops represent autoregressive effects. Line thickness and color intensity reflect the magnitude of the regression coefficient; nonsignificant edges (FDR-adjusted *p* > .05) are pruned.

**Table 4.** Cross-lagged temporal network for OM meditation

		Source							
		Temporal theta	Frontal theta	Posterior theta	Frontal alpha	Temporal alpha	Posterior alpha	SMS-Mind	SMS-Body
		*β* (*SE*)	*β* (*SE*)	*β* (*SE*)	*β* (*SE*)	*β* (*SE*)	*β* (*SE*)	*β* (*SE*)	*β* (*SE*)
Targets	Temporal theta	**0.79 (0.23)** **	−0.02 (0.2)	0.11 (0.18)	−0.34 (0.27)	0.12 (0.18)	0.27 (0.17)		
	Frontal theta	0.24 (0.23)	**0.41 (0.19)** *	0.21 (0.18)	−0.40 (0.27)	<0.01	**0.43 (0.18)** *		
	Posterior theta	0.12 (0.23)	−0.07 (0.2)	**0.85 (0.18)** **	−0.29 (0.27)	0.08 (0.18)	0.23 (0.17)		
	Front alpha	0.03 (0.23)	0.01 (0.2)	−0.01 (0.18)	0.31 (0.28)	0.23 (0.19)	**0.37 (0.17)** *		
	Temporal alpha	−0.01 (0.24)	0.10 (0.2)	−0.05 (0.18)	−0.34 (0.28)	**1.02 (0.19)** **	0.23 (0.17)		
	Posterior alpha	0.05 (0.24)	0.09 (0.2)	−0.10 (0.18)	−0.34 (0.28)	0.18 (0.19)	**1.07 (0.17)** **		
	SMS-Mind	**−0.64 (0.25)** **	0.07 (0.19)	0.24 (0.2)	−0.03 (0.29)	0.40 (0.21)	0.02 (0.2)	**0.31 (0.06)** **	0.08 (0.06)
	SMS-Body	−0.38 (0.24)	0.03 (0.18)	0.2 (0.19)	**0.59 (0.28)** *	−0.01 (0.2)	**−0.42 (0.19)** *	−0.02 (0.07)	**0.47 (0.06)** **

*Note*. Significant paths are **bolded** for ease of reference. Significance testing is not conducted for *β* values < 0.01.

* *p* < 0.05.

** *p* < 0.01.

However, similar to FA, the cross-lagged OM network was characterized by robust positive autoregressive effects for both mindfulness subscales. Again, higher SMS-Mind scores predicted higher SMS-Mind in the following session (*β* = 0.31, *SE* = 0.06, *p* < .001), as did SMS-Body scores (*β* = 0.47, *SE* = 0.06, *p* < .001). This consistency across both meditation types further supports the interpretation of these autoregressive links as an indicator that training is taking place, insofar that the ability to cultivate a mindful state during one session appears to strengthen mindfulness during subsequent sessions, even after accounting for neural influences.

Centrality measures for the OM cross-lagged network (seeFigure 7) again revealed that subjective state mindfulness as the central recipient of the network’s predictive effects (SMS-Body: InStrength = 1.30, SMS-Mind: InStrength = 0.76). Reinforcing its role as a key hub across meditation types, posterior alpha once again emerged as the network’s singular driver of change, with an OutStrength (2.24) dramatically higher than any other node. Its influence extended even to other neural nodes, predicting activity in frontal alpha (*β* = 0.37, *SE* = 0.17, *p* = .03) and frontal theta (*β* = 0.43, *SE* = 0.18, *p* = .02). Yet, it is worth noting that in contrast to the FA network, posterior alpha exerted a selective influence on SMS-Body but not SMS-Mind, a network distinction that appears to highlight the unique interoceptive characteristics of OM. This configuration highlights posterior alpha as a critical modulator of training across both types of meditation, but with its influence being selectively directed toward the development of bodily awareness within OM.

**Figure 7.**
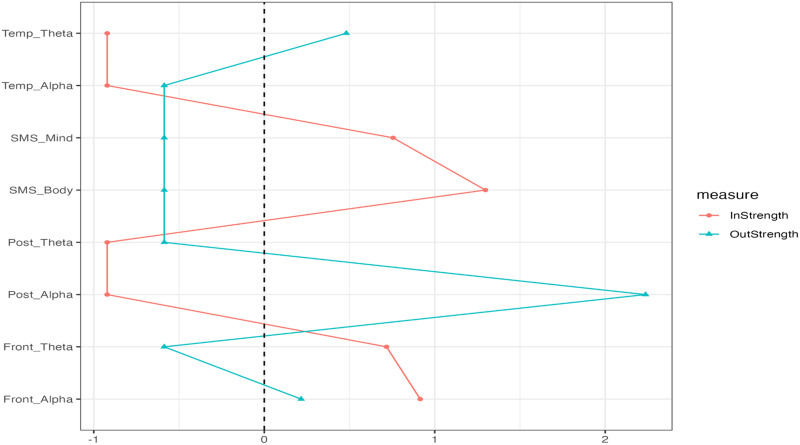
OM cross-lagged temporal network centrality metrics. Plot displaying centrality indices for the OM cross-lagged network. InStrength (red circle) quantifies the sum of incoming edge weights, reflecting the extent to which a node is influenced by others over time. OutStrength (blue triangle) quantifies the sum of outgoing edge weights, reflecting a node’s influence on the future state of the network. Centrality indices are calculated based on standardized partial directed correlations, which differ from the regression coefficients reported in Table 4.

#### Contemporaneous network.

Similar to the FA network, the contemporaneous OM network (seeFigure 8 andTable 5), which likewise demonstrated excellent stability (mean proportion of replicated bootstrapped samples = 0.98), revealed a strong correlation between SMS-Mind and SMS-Body (*β* = 0.51, *SE* = 0.05, *p* < .001). While not statistically significant, it is worth noting that decreased posterior alpha power trended toward an association with increased SMS-Body (*β* = −0.12, *SE* = 0.06, *p* = .06), providing a potentially important point of dissociation from the FA network, where a negative link between frontal theta and SMS-Mind was observed. This possibility is further supported by the centrality analysis (seeFigure 9), which revealed posterior alpha (betweenness = 0.94, closeness = 0.78, strength = 0.65) and frontal alpha (betweenness = 0.94, closeness = 1.56, strength = 0.62) as key hubs within the contemporaneous network.

**Figure 8.**
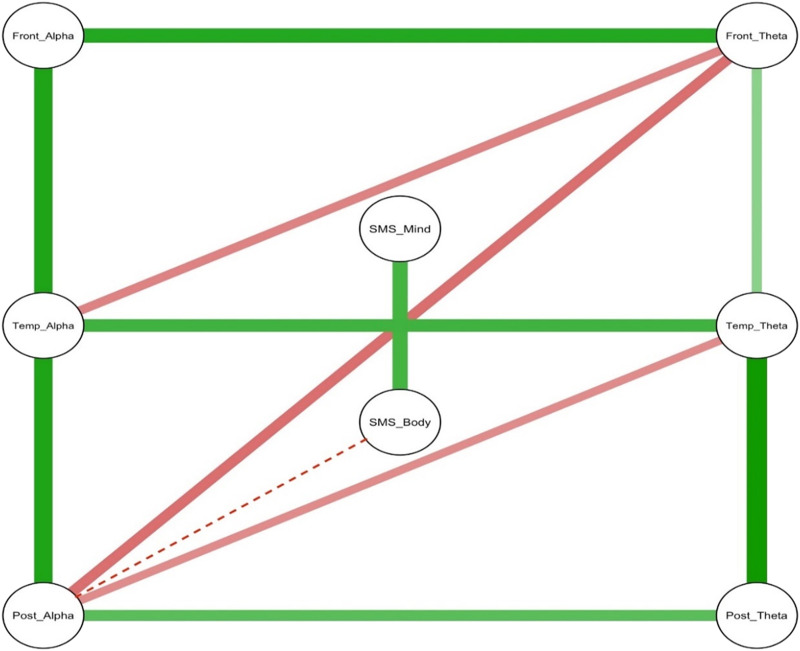
OM contemporaneous network. Graphical depiction of significant within-session partial correlations linking region-specific EEG spectral power (alpha, theta) and subjective state mindfulness ratings (SMS-Mind, SMS-Body) during OM practice. Edges represent undirected associations between variables during the concurrent session *t*. Green edges denote positive partial correlations; red edges denote negative partial correlations. The dashed red edge represents a marginally significant (*p* < .10) association between posterior alpha and SMS-Body included for theoretical relevance. Line thickness and color intensity reflect the magnitude of the association; nonsignificant edges (FDR adjusted *p* > .05) are pruned.

**Table 5.** Contemporaneous network for OM meditation

		Source						
		Temporal theta	Frontal theta	Posterior theta	Frontal alpha	Temporal alpha	Posterior alpha	SMS-Mind
		*β* (*SE*)	*β* (*SE*)	*β* (*SE*)	*β* (*SE*)	*β* (*SE*)	*β* (*SE*)	*β* (*SE*)
Targets	Frontal theta	**0.31 (0.09)** **						
	Posterior theta	**0.69 (0.05)** **	**0.31 (0.08)** **					
	Frontal alpha	−0.11 (0.11)	**0.60 (0.06)** **	−0.17 (0.11)				
	Temporal alpha	**0.52 (0.08)** **	**−0.34 (0.09)** **	−0.19 (0.12)	**0.53 (0.09)** **			
	Posterior alpha	**−0.31 (0.11)** **	**−0.39 (0.09)** **	**0.45 (0.10)** **	**0.62 (0.08)** **			
	SMS-Mind	−0.03 (0.06)	−0.05 (0.06)	−0.04 (0.06)	−0.07 (0.06)	0.24 (0.13)	0.08 (0.06)	
	SMS-Body	−0.08 (0.06)	−0.02 (0.06)	0.08 (0.06)	0.09 (0.06)	0.05 (0.06)	−0.12 (0.06)	**0.51 (0.05)** **

*Note*. Significant paths are **bolded** for ease of reference. Significance testing is not conducted for *β* values < 0.01.

* *p* < 0.05.

** *p* < 0.01.

**Figure 9.**
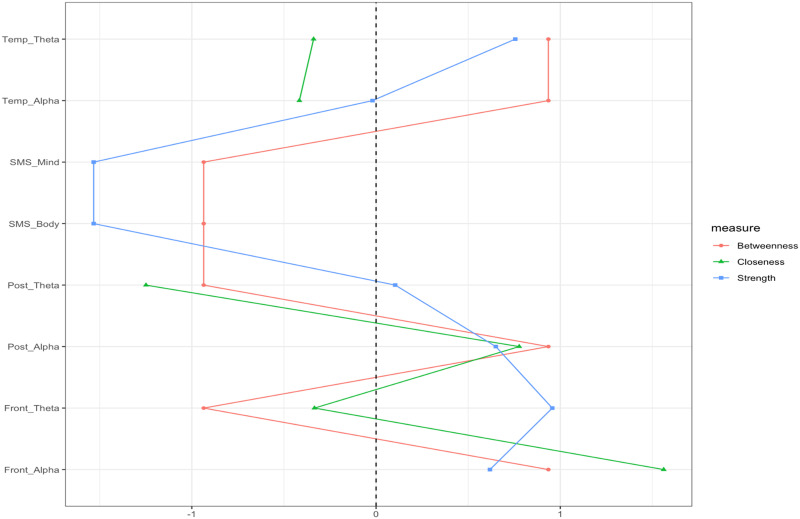
OM contemporaneous network centrality metrics. Plot displaying centrality indices for the OM contemporaneous network. Strength (blue square) represents the sum of absolute edge weights connected to a node, indicating its overall level of direct connectivity. Closeness (green triangle) reflects the inverse of the sum of shortest distances to all other nodes. Betweenness (red circle) indicates how often a node lies on the shortest path between two other nodes. Higher values indicate greater influence within the network.

## DISCUSSION

The primary aim of the current study was to elucidate the dynamic relationship between neural oscillatory activity and subjective experience during meditation training, thereby directly addressing the historical disconnect between the establishment of neural markers (i.e., alpha and theta power) elicited by state mindfulness, and linking them directly with the subjective quality of state mindfulness itself. Furthermore, by applying longitudinal network analysis to a time-intensive, within-subject dataset comparing FA and OM, we sought to move beyond the static limitations of prior cross-sectional and pre–post designs (Cahn & Polich, 2006;Lee et al., 2018;Lomas et al., 2015) to instead characterize the cross-lagged and contemporaneous influence of region-specific patterns of alpha and theta power on state mindfulness quality. The findings provide direct evidence that neural activity occurring during meditation training both prospectively and concurrently influenced subjective mindful awareness of mind and body. Moreover, the network structures of these relationships exhibit fundamental similarities, yet also key distinctions between FA and OM, thereby providing robust, data-driven support for the long-held theoretical separation between these core practices (Lutz et al., 2008,^2015^), while significantly advancing mechanistic understanding of the key brain networks that most strongly influence mindfulness.

### Shared Network Properties of Training Development Across FA and OM

A foundational similarity emerged across both FA and OM networks, which revealed robust positive autoregressive effects for both SMS-Mind and SMS-Body, independent of neural influences. This demonstrates a powerful session-to-session carryover effect, where higher levels of mindfulness reported during one practice session strongly predicted greater mindfulness during subsequent sessions, providing compelling empirical support for the fundamental conceptualization of mindfulness as a trainable skill via repeated meditation practice. Importantly, this dynamic carryover pattern converges with complementary linear mixed-effects analyses showing modest but reliable increases in mean SMS scores, indicating mindfulness development may be characterized by both gradual mean-level improvement and prospective session-to-session influences. Indeed, the consistency observed across both meditation types lends further credence to the idea that a general process of skill development is occurring over the course of training.

Beyond this shared dynamic, the specific network structures for FA and OM revealed a strikingly common pattern of opposing regional neural influences that fundamentally challenge conventions that monotonic increases or decreases in alpha and theta power uniformly reflect enhanced or reduced mindfulness. Most prominently, alpha power exhibited a clear frontal–posterior dissociation across both meditation types, such that greater frontal alpha power prospectively enhanced subsequent mindfulness quality, while increased posterior alpha power exerted an opposing inhibitory effect. The facilitative influence of frontal alpha aligns with its established role in maintaining top-down control of selective attention and suppressing task-irrelevant information (Berger & Davelaar, 2018;Klimesch, 2012;Misselhorn et al., 2019). In contrast, the inhibitory effect of posterior alpha incisively demonstrates that not all increased alpha activity is associated with greater mindfulness or enhanced cognition, but may rather signal attentional lapses, mind wandering, or reduced vigilance (Hanslmayr et al., 2005;Jin et al., 2019;Molina et al., 2019;O’Connell et al., 2009).

A similar opposing pattern was observed for theta power, which showed that greater posterior theta power prospectively enhanced mindfulness during FA, whereas decreased temporal-central theta power was predictive of a subsequent increase in state mindfulness quality across both FA and OM practice. The facilitative influence of posterior theta challenges the frontal-centric view of attentional control in FA meditation, where frontal midline theta has been the predominant focus (Brandmeyer & Delorme, 2013,^2020^). Instead, our finding aligns with work demonstrating that experienced meditators exhibit higher theta power and a shifted distribution of activity toward posterior regions at rest (McQueen et al., 2024) and that parietal theta activity is associated with heightened attention in real-world interactive tasks (Kaushik et al., 2022).

The inhibitory influence of temporal-central theta evidenced across both meditation types further reinforces the role of regional specificity in determining the nature of the relationship between neural oscillatory activity and subjective mindfulness quality. Although speculative, this inhibitory effect may reflect increased demands on cognitive control, insofar that activity within temporal-central and nearby frontal regions have been shown to be implicated in mental effort, conflict monitoring, and exertion of cognitive control more broadly (Brilliant et al., 2024;Cavanagh & Frank, 2014;Eisma et al., 2021;McFerren et al., 2021;Tan et al., 2024). Consequently, sessions that are dominated by higher demands on control may be marked by continuous management of distraction and mind wandering, a process that may lead to lower self-reported mindfulness.

### Distinguishable Network Features Between FA and OM

Although both meditation types exhibited similar patterns of regional influence, their network structures revealed theoretically meaningful differences that support their unique neurophenomenological profiles. The cross-lagged FA network revealed greater interconnectivity, with neural nodes influencing both mindfulness of mind and body. This dynamic interplay is accentuated by the network’s centrality metrics, where SMS-Mind and SMS-Body exhibited the highest InStrength, positioning subjective experience as the primary downstream target of the network’s temporal influences. Conversely, posterior alpha displayed the highest OutStrength, identifying it as the key neural driver of session-to-session change. This configuration provides strong evidence for a system where the primary role of meditation-related neural activity is to prospectively shape the phenomenology of meditation and the process of cultivating mindful awareness. The contemporaneous FA network provides further convergent support, where frontal theta was negatively associated specifically with concurrent SMS-Mind scores, identifying a reduction of frontal theta as a neural*correlate* linked to enhanced mindfulness of mental activity—a role underscored by its central position as the most influential node within the network.

The network structure of OM, although containing parallels to the effects observed in FA, revealed theoretically meaningful distinctions. Most critically, the frontal-posterior alpha dissociation was observed exclusively for SMS-Body scores, providing compelling, data-driven evidence linking OM meditative neural activity with the development of interoceptive awareness, a core theoretically defining feature of OM practice (Lutz et al., 2008). Functionally, the same underlying frontal-posterior attentional dynamic is recruited during OM, but directed specifically toward bodily states.

The centrality metrics further illuminate this distinction. While posterior alpha once again emerged as the dominant driver of session-to-session change with the highest OutStrength (mirroring its key role in FA), its downstream influence appeared more selective to SMS-Body, which exhibited the highest InStrength. The contemporaneous network reinforced this pattern, revealing a marginal but selective negative association between posterior alpha and concurrent SMS-Body scores, paralleling the unique negative association between frontal theta and SMS-Mind observed in the FA network.

### Methodological Advances and Implications for Basic and Translational Science

The present findings advance mechanistic understanding of FA and OM meditation by elucidating the dynamic and contemporaneous patterns through which neural oscillatory activity influences subjective mindfulness quality. Illustratively, the opposing facilitative versus inhibitory dynamic patterns observed across both FA and OM may reflect a common neural signature of meditation training among novices—the recurring “push and pull” between periods of concentrated awareness and the cognitively demanding process of identifying and overcoming distraction. This central finding is consistent with prevailing perspectives that view meditation as an ongoing negotiation between neurocognitive systems supporting sustained attention and performance monitoring processes involved in goal maintenance and attentional reorientation (Ganesan et al., 2022;Hölzel et al., 2011;Lutz et al., 2008).

On the other hand, the contemporaneous networks revealed distinct neural correlates specific to each practice. For FA, reduced frontal theta power was selectively associated with higher state mindfulness of mind, whereas for OM, a similar (although marginal) negative association was observed between posterior alpha and state mindfulness of body. Indeed, a major strength of our longitudinal network approach lies in the ability to disentangle the aforementioned cross-session “training” effects from these within-session “state” relationships. The value of this analytic separation becomes evident when comparing these findings to our prior work using a static state induction design (White et al., 2025). Although both studies identified a negative association between posterior alpha and state mindfulness during OM (suggesting that it may be a robust state correlate), the negative frontal theta relationship in FA was present only in the present analysis. This divergence highlights how intensive repeated sampling can reveal unique contemporaneous brain–mind relationships that may emerge solely after an extended period of training. Critically, this analytic separation is achieved parsimoniously within a single statistical model, allowing for a more data-driven interpretation that distinguishes the neural*mechanisms* that drive skill development over time (cross-lagged effects) from the neural*correlates* that characterize the quality of a given meditative state (contemporaneous effects). Moreover, because regionally differentiated alpha and theta power are included as distinct nodes within the same network, the model simultaneously accounts for their unique contributions, clearly demonstrating that activity across both frequency bands exerts*independent* influence on the quality of state mindfulness.

This ability to elucidate key network features is sharpened by the centrality analyses. The consistent emergence of posterior alpha as the node with the highest OutStrength in the cross-lagged networks, for example, positions it as a critical driver of training-related change across both FA and OM. Identifying such influential nodes is a critical step toward advancing the translational neuroscience of mindfulness, providing a data-driven roadmap that can be used to develop targeted neuromodulation or neurofeedback programs. Rather than employing “monotonic” strategies to broadly increase or decrease spectral power, our findings inform a more sophisticated path forward.

For instance, our results generate directly testable hypotheses for causal interventions like transcranial alternating current stimulation (tACS). Specifically, our findings suggest that applying tACS to posterior regions to entrain alpha may disrupt mindfulness quality, while suppressing it could enhance state mindfulness. Moreover, the opposing frontal-posterior dynamic introduces the potential for synergistic, multisite protocols that simultaneously enhance frontal alpha while suppressing posterior alpha. This level of regional and functional specificity paves the way for data-informed protocols designed to optimize training outcomes by directly modulating the core neural dynamics of practice.

Finally, while the network structures identified here offer a nuanced group-level model, perhaps the most promising future direction lies in applying this framework to individuals. The intensive, repeated-sampling design of the ILM protocol is uniquely suited for generating idiographic or “N-of-1” networks, provided sufficient data are collected from a single participant. Such an approach would allow for the identification of neural dynamics, the specific facilitative or inhibitory drivers, which are most influential for a given individual. This aligns directly with the core tenets of precision neuroscience, which aims to tailor interventions based on individual-level data rather than group averages (Collins & Varmus, 2015;Hernández et al., 2024;Poldrack, 2017). Ultimately, a practitioner’s unique neurophenomenological network could serve as a personalized roadmap, guiding the development of neurofeedback or neuromodulation protocols tailored not just to a specific practice, but to the person engaging in it (Brandmeyer & Reggente, 2023).

### Limitations and Future Directions

While the current study exhibits notable strengths through its time-intensive sampling design and novel application of longitudinal network analysis, it is not without key limitations, several of which inspire promising directions for future research. First, the longitudinal network models employed here necessarily assume structuralstationarity, such that cross-lagged contemporaneous relationships between neural activity and subjective mindfulness are estimated as constant across the 8-week training period. Although the multilevel GVAR framework separates within- from between-subject variance, we did not explicitly detrend the time series or test for time-varying network parameters. Consequently, gradual training-related changes to the network coupling structure may not be fully captured by the present models. Future work involving higher-density time series could relax this assumption by using detrending procedures or time-varying VAR approaches.

Relatedly, we constrained cross-lagged effects to model the prospective influence of neural activity on subsequent subjective experience to maintain parsimony and interpretability. However, the neurophenomenological relationship between neural dynamics and subjective experience is very likely reciprocal, and thus, future studies involving larger samples or long time series could therefore leverage unconstrained or bidirectional models to directly examine feedback processes between mind and brain across training.

Second and perhaps most saliently, our analyses were limited to examining spectral power in only the alpha and theta bands, omitting investigation of other potentially important frequency ranges that have likewise been implicated in FA and OM meditation (e.g., beta, gamma, and delta;Cahn & Polich, 2006;Lee et al., 2018;Lieberman et al., 2025). Although we intentionally elected to focus on alpha and theta due to their historical prominence within the contemplative neuroscience literature, the ILM dataset nevertheless provides ample opportunity to extend our network models to explore activity within other frequency ranges.

Similarly, our EEG analysis relied on traditional spectral decomposition methods using fixed canonical frequency bands, rather than leveraging newer, more sophisticated analytic techniques (seeLin et al., 2025, for a review of emerging methods). For example, recent methodological advances suggest that changes in broadband power can be driven by shifts in individual peak oscillatory frequency or by changes in the aperiodic (1/*f*) component of the EEG signal (Donoghue, Dominguez, et al., 2020). Consequently, applying spectral parameterization tools (e.g., fitting oscillations and over f;Donoghue, Haller, et al., 2020;Gerster et al., 2022) would permit a more nuanced interrogation of the observed effects to determine the extent to which the neural influences on subjective mindfulness quality are driven by true oscillatory activity from these other contributing factors. Absent such decomposition, interpretations linking observed alpha and theta patterns to specific oscillatory functions or mechanisms, including the suggestions made above, should be regarded as tentative. Similarly, our data are amenable to the computation of nonlinear measures such as neural complexity (Atad et al., 2023) and criticality (Irrmischer, Houtman, et al., 2018;Irrmischer, Poil, et al., 2018), enabling tests of whether their session-to-session variability relates to state mindfulness. Integrating these advanced spectral and nonlinear measures into our longitudinal network framework holds significant potential to further enrich understanding of the brain dynamics that support meditation progress and mindfulness skill acquisition.

Regarding our EEG data collection methods, we used a 32-channel array, a setup intentionally chosen to balance spatial resolution with the practical demands of a time-intensive longitudinal protocol. Our findings are therefore based on scalp-level potentials extracted from a relatively limited number of electrode sites, which are inherently susceptible to volume conduction and thus have limited precision for resolving the underlying neural generators of observed activity (Nunez & Srinivasan, 2006;Rutkove, 2007). Moreover, although our partitioning of electrodes into frontal, temporal-central, and posterior regions was deliberate to match prior research (Lagopoulos et al., 2009;Lin et al., 2020), these broad groupings may fail to capture more localized or distributed patterns of activity. Consequently, future studies employing denser arrays (e.g., 64 or 128 channels) represent promising, albeit logistically ambitious, next steps. The higher spatial resolution afforded by such arrays would not only allow for the identification of more distributed scalp divisions but would also position future work to localize network effects using source localization techniques (Michel & Brunet, 2019). While source analysis remains a valuable future direction for our dataset, denser arrays will be needed to achieve the highest level of spatial precision.

Beyond these methodological considerations, the scope of our investigation was circumscribed to FA and OM meditation. Our research framework, however, is highly adaptable and could be applied to characterize the network properties of other contemplative practices, such as loving-kindness, nondual awareness, or visualization-based meditation. Similarly, our reliance on the SMS as the primary subjective outcome, while psychometrically sensible for an initial investigation, represents just one possible method to capture meditation experience. Future research could incorporate more granular or multidimensional experience-sampling measures to capture specific phenomenal features of meditation states (e.g., aperture or stability of attention, vigilance, effort, affective valence/arousal;Lutz et al., 2015). Pairing our network modeling approach with detailed subjective self-report measures of meditation quality could provide a richer window into the dynamic brain–mind relationships that characterize different meditation practices and traditions. Finally, our modest sample size (*N* = 16), although demographically diverse, favors data density (i.e., sessions per participant) over the total number of participants, thus limiting the generalizability of the network structures and highlighting the need for future replication involving larger cohorts.

Taken together, the current study represents an important, albeit initial, step. By pairing a time-intensive sampling design with longitudinal network analysis, we demonstrate a feasible and potentially fruitful way to map the dynamic interplay between meditation-specific neural activity and subjective experience over the course of repeated training. The true value of this framework lies not only in the specific findings reported here but also in its capacity to be systematically enriched by pursuing the exciting future directions discussed, from incorporating advanced EEG analytic methods to the inclusion of different meditation practices and more granular subjective reports. Toward this end, we hope this work serves as a useful foundation and invitation for other researchers to build upon our data and methods, collectively advancing our understanding of the neurophenomenology of contemplative practice.

## AUTHOR CONTRIBUTIONS

Yanli Lin: Conceptualization; Data curation; Formal analysis; Funding acquisition; Investigation; Methodology; Project administration; Supervision; Writing – original draft; Writing – review & editing. Marne White: Data curation; Formal analysis; Investigation; Methodology; Project administration; Visualization; Writing – review & editing. Jihong Zhang: Formal analysis; Methodology; Software; Visualization; Writing – review & editing. Todd Braver: Conceptualization; Funding acquisition; Investigation; Project administration; Resources; Supervision; Writing – review & editing.

## FUNDING INFORMATION

Yanli Lin, WUSTL McDonnell Center for Systems Neuroscience, Award ID: SGP Award. Yanli Lin, National Institute on Aging (https://dx.doi.org/10.13039/100000049), Award ID: F32-AG069499. Yanli Lin, University of Arkansas Libraries Open Access Publishing Fund.

## DATA AVAILABILITY

All data and analytic code, including full model specifications and additional visualizations, are publicly available on the OSF at https://osf.io/buxah/. For transparency, we note that the longitudinal network approach represents a methodological deviation from our preregistered aims (https://osf.io/gws3q), which specified the use of linear mixed-effects models. This deviation was made based on the theoretical and empirical considerations outlined in the manuscript.

## TECHNICAL TERMS

Focused attention (FA): A meditation practice emphasizing sustained selective attention on a chosen target object (e.g., the breath), with attention redirected when mind wandering or distraction is detected.
Open monitoring (OM): A meditation practice fostering nonreactive, nonjudgmental awareness of present momentary experience without focusing on a single object.
Neurophenomenology: A research framework integrating objective physiological measurement with systematic first-person reports of subjective experience to map brain–mind relationships.
Graphical vector autoregression (gVAR): A statistical framework for estimating dynamic networks from time-series data by modeling both temporal (lagged) and same-time (contemporaneous) relationships among variables.
Autoregressive effect: The predictive relationship of a variable on itself over time; a measure of inertia.
Contemporaneous network: A network representing undirected partial correlations among variables measured at the same time point, reflecting associations after accounting for temporal effects.
Cross-lagged (temporal) network: A directed network representing how variables at one time point (e.g., *t* − 1) predict other variables at a subsequent time point (e.g., *t*).
Stationarity: The assumption that the key statistical properties of a time series (e.g., mean, variance, relationship between variables) remain stable over time.
